# A national case-crossover analysis of the short-term effect of PM_2.5_ on hospitalizations and mortality in subjects with diabetes and neurological disorders

**DOI:** 10.1186/1476-069X-13-38

**Published:** 2014-05-22

**Authors:** Antonella Zanobetti, Francesca Dominici, Yun Wang, Joel D Schwartz

**Affiliations:** 1 Department of Environmental Health, Harvard School of Public Health, Boston, MA, USA; 2 Department of Biostatistics, Harvard School of Public Health, Boston, MA, USA

**Keywords:** PM_2.5_, Diabetes, Neurological disorders, Mortality risk, Hospitalizations

## Abstract

**Background:**

Diabetes and neurological disorders are a growing burden among the elderly, and may also make them more susceptible to particulate air matter with aerodynamic diameter less than 2.5 μg (PM_2.5_). The same biological responses thought to effect cardiovascular disease through air pollution-mediated systemic oxidative stress, inflammation and cerebrovascular dysfunction could also be relevant for diabetes and neurodegenerative diseases.

**Methods:**

We conducted multi-site case-crossover analyses of all-cause deaths and of hospitalizations for diabetes or neurological disorders among Medicare enrollees (>65 years) during the period 1999 to 2010 in 121 US communities. We examined whether 1) short-term exposure to PM_2.5_ increases the risk of hospitalization for diabetes or neurological disorders, and 2) the association between short-term exposure to PM_2.5_ and all-cause mortality is modified by having a previous hospitalization of diabetes or neurological disorders.

**Results:**

We found that short term exposure to PM_2.5_ is significantly associated with an increase in hospitalization risks for diabetes (1.14% increase, 95% CI: 0.56, 1.73 for a 10 μg/m^3^ increase in the 2 days average), and for Parkinson’s disease (3.23%, 1.08, 5.43); we also found an increase in all-cause mortality risks (0.64%, 95% CI: 0.42, 0.85), but we didn’t find that hospitalization for diabetes and neurodegenerative diseases modifies the association between short term exposure to PM_2.5_ and all-cause mortality.

**Conclusion:**

We found that short-term exposure to fine particles increased the risk of hospitalizations for Parkinson’s disease and diabetes, and of all-cause mortality. While the association between short term exposure to PM_2.5_ and mortality was higher among Medicare enrollees that had a previous admission for diabetes and neurological disorders than among Medicare enrollees that did not had a prior admission for these diseases, the effect modification was not statistically significant. We believe that these results provide useful insights regarding the mechanisms by which particles may affect the brain. A better understanding of the mechanisms will enable the development of new strategies to protect individuals at risk and to reduce detrimental effects of air pollution on the nervous system.

## Background

There is clear evidence that long- and short-term exposures to particulate air pollution are associated with both acute and chronic mortality risk; multi-site studies have shown that ambient particulate pollution is associated with increased risk of deaths or hospitalizations for broadly defined cardiovascular disease [1-9]. Several studies have also reported associations between short-term particulate matter (PM_2.5_) exposure and cerebrovascular disease and stroke [10-15]. Two studies examined the association between pollution and mortality [16] or admission [12] separately by stroke type and found that pollution was associated with ischemic but not hemorrhagic stroke.

The mechanisms responsible for the effects of pollution on cerebrovascular disease and stroke are not fully understood, but could be mediated through a combination of autonomic, hemostatic, inflammatory, and vascular endothelial disturbances with consequent changes in cardiac and vascular function. For example some studies have suggested that particulate air matter with aerodynamic diameter less than 2.5 μg (PM_2.5_) may be associated with increased blood pressure, increased peripheral vascular resistance, decreased brachial artery diameter, and decreased brachial artery flow–mediated dilation [17-23]. Other studies found that PM_2.5_ may be associated with increased C-reactive protein [24,25]. PM_2.5_ and its components have also been shown to increase oxidative stress, [24,26-28] to decrease plaque stability [29,30], and to increase atherosclerosis [31]. Derangements in cerebral vascular function have been associated with stroke incidence, as well as cognitive impairment, dementia, and depression. Vascular oxidative stress and inflammation are key pathogenic factors in neurovascular dysfunction [32]. The endothelial dysfunction induced by oxidative stress can release vascular endothelial growth factor and prostanoids, which promote vascular leakage, protein extravasation, and cytokine production [32].

Given the documented associations between exposure to ambient particulate air pollution and cerebrovascular events, peripheral vascular function, cerebral vascular function [33,34], but also oxidative stress and inflammation, we can hypothesize that PM_2.5_ may also affect neurological disorders such as dementia, Alzheimer’s and Parkinson’s. Air pollution exposure has been hypothesized to increased inflammation in the brain [35], and brain inflammation has been implicated in the development of Alzheimer’s disease (AD) [36]. Therefore the same biological responses thought to effect cardiovascular disease through air pollution-mediated systemic oxidative stress and inflammation could also be relevant for diabetes and neurodegenerative diseases [15,37].

Two recent studies have reported associations of traffic with lower cognitive function in elderly men [38] and with older women in the Nurses’ Health Study [39]. Several epidemiological studies have shown that people with diabetes are vulnerable to cardiovascular health effects associated with exposure to particle air pollution [20,40-43]. People with diabetes are at greater risk for acute environmental perturbations of vascular function because of chronic endothelial dysfunction, autonomic dysregulation, atherosclerosis, or dysregulation of fluid balance and the renin-angiotensin system [44,45]. Hence neurological disorders and diabetes are plausible targets for PM_2.5_.

The elderly represent a particularly susceptible population, and the growth in the number and proportion of older adults in the United States is unprecedented. Diabetes, cognitive decline, Parkinson’s disease, dementia, and Alzheimer's disease are also a growing burden. Alzheimer’s disease is the most common cause of dementia among older people. Estimates vary, but experts suggest that as many as 5.1 million Americans may have AD, while in 2006 there were 26.6 million cases worldwide. Dementia is the loss of cognitive functioning and memory, vascular dementia is due to many small strokes. Alzheimer’s disease (AD) is an irreversible, progressive brain disease that slowly destroys memory, thinking, and behavior. Parkinson's disease is a disorder of the brain that leads to shaking (tremors) and difficulty with walking, movement, and coordination; it can occur when the nerve cells in the brain that make dopamine are slowly destroyed, resulting in impairment of muscular control by that neurotransmitter. Parkinson's disease has also been related to autonomic dysfunction. Multiple sclerosis (MS) is an autoimmune disease that affects the brain and spinal cord, and it is caused by nerve damage which in turn can be caused by inflammation. For at least 20 years, diabetes rates in North America have been increasing substantially. The increasing prevalence of diabetes makes this susceptibility factor especially important to study.

The hypotheses of this study are: 1) short term exposure to PM_2.5_ might be associated with an increased risk of hospitalizations for diabetes or neurological disorders in the elderly; and 2) elderly with diabetes or neurological disorders may have higher risk of deaths from short term exposure to PM_2.5_ compared to elderly without these conditions. Thus, the purpose of this investigation is twofold: we will estimate the effect of short-term exposure of PM_2.5_ on 1) hospitalizations for neurological disorders and diabetes, and 2) on all-cause mortality in all Medicare deaths. Importantly we will also assess whether the mortality risks associated with PM_2.5_ among the elderly are modified by a previous hospital admission for a neurological disorder or diabetes.

To achieve these aims we will conduct a multi-site case-crossover study, where for each county, we estimate: 1) the short-term effects of PM_2.5_ on hospitalizations for neurological disorders and diabetes; 2) the short-term effects of PM_2.5_ on all-cause mortality; 3) and the short-term effects of PM_2.5_ on all-cause mortality in a susceptible population defined as persons 65 or older with at least one Medicare covered hospital admission for diabetes or neurological disorders.

## Methods

### Health data

We used the Medicare beneficiary denominator file from the Centers for Medicare and Medicaid services (CMS) to identify beneficiaries who were enrolled in the Medicare fee-for-service (FFS) plan between 1999 and 2010. The denominator file contains information on beneficiaries’ eligibility and enrollment in Medicare and the date of death was cross-referenced with the Social Security Administration's Master Beneficiary Record [46].

From the initial denominator file we excluded beneficiaries with age < 65 years, those enrolled in managed-care programs over an entire year, and those who resided outside of the targeted counties. We identified all deaths by selecting those subjects who died among all Medicare enrollees denominator file.

We linked the person-years beneficiary data with the Medicare Provider Analysis and Review (MEDPAR) inpatient data to identify all Medicare FFS patients who were hospitalized for the targeted medical conditions. The MEDPAR inpatient data includes information on patient demographics (age, sex, race), dates of admission and discharge, admission sources and types, principal and secondary diagnosis codes, and procedure codes, defined by the International Classification of Diseases, Ninth Revision, (ICD-9).

We used the Medicare claims records from the Medicare Provider Analysis and Review (MEDPAR) file to select only emergency admissions, and to identify previous cause of hospital admissions, based on primary and secondary diagnosis codes of all hospitalizations for any causes up to one year prior to the initial condition-specific hospitalization. Because diabetes and neurological disorders might not be the primary reason for an admission, individuals for whom the condition was noted as the primary admission cause or as any of nine coexisting conditions were classified as having the condition for the mortality analysis. We excluded patients who could not be merged with the Medicare denominator file.

The medical conditions that we examined were: diabetes (ICD-9: 250), dementia (ICD-9: 290), Alzheimer's disease (ICD-9: 331.0), Parkinson's disease (ICD-9: 332), and multiple sclerosis (ICD-9: 340). The primary code of admission reflects the main cause of admissions and therefore we used only primary emergency admissions to study the effect of PM_2.5_ on hospitalization.

The research was approved by the institutional review boards (IRB) of the Harvard School of Public Health.

### Environmental data

We obtained particulate air matter with aerodynamic diameter less than 2.5 μg (PM_2.5_) data from US Environmental Protection Agency’s Air Quality System Technology Transfer Network (U.S. EPA Technology Transfer Network, 2005) which provide daily PM_2.5_ concentrations from the U.S. EPA’s National and State Local Ambient Monitoring stations (NAMS and SLAMS).

For most cities, the metropolitan county encompassed the city and much of its suburbs; therefore we aggregated counties where the city’s population extends beyond the boundaries of one county; henceforth we refer to the analyzed geographical areas as “community”.

Given that PM_2.5_ is not available daily in every city, to be included in our study, we required that at least 219 (60% of days) days of data in at least one year be available in each community. With this criterion, we selected 121 communities (180 counties) across the US for the years 1999–2010, based on the availability of air pollution, and representing a geographic distribution across the country.

Many of the communities have more than one monitoring location, requiring a method to average over multiple locations. Therefore we computed the community daily mean PM_2.5_ concentrations using an algorithm previously described [47,48] that accounts for the different monitor-specific means and variances.

We defined our exposure as the 2 days average of PM_2.5_, that is, the average of the same (lag 0) and previous (lag 1) day. We present the results as percent change in mortality (or cause specific hospital admission) for 10 μg/m^3^ increase in the two days average of PM_2.5_.

We obtained local meteorological data from the National Oceanic and Atmospheric Administration (NOAA), including daily temperature (mean, min, max), daily dew point, date, and weather station.

### Analytical strategy

To estimate the short-term effects of PM_2.5_ on hospitalizations for neurological disorders and diabetes, and to determine whether the mortality risks associated with short-term PM_2.5_ among elderly is exacerbated by a previous hospital admission for neurological disorders or diabetes, we conducted a community specific case-crossover analysis.

We created indicator variables denoting whether or not an individual had a primary or secondary hospitalization for diabetes or neurological disorder at any time before death. In the model having as outcome all-cause deaths, we also examined effect modification by specific cause of prior admission by including an interaction terms between PM_2.5_ and these indicator variables in the same model.

The case-crossover design was developed as a variant of the case–control design to study the effects of transient exposures on acute events [49]. This design samples only cases and compares each subject’s exposure experience in a time period just prior to a case-defining event with that subject’s exposure at other times. Since there is perfect matching on all measured or unmeasured subject characteristics that do not vary over time there can be no confounding by those characteristics.

Bateson and Schwartz [50,51] demonstrated that by choosing control days close to event days, even very strong confounding of exposure by seasonal patterns could be controlled by design in the case control approach. This makes the approach an attractive alternative to the Poisson models for time series analysis.

We applied the time-stratified case-crossover design, and we defined the case as the day of death (or hospitalization); we then chose control days as every third day in the same month and year as the case.

The analysis was conducted for the hospitalization data and for the mortality data for each community separately, and we applied a conditional logistic regression controlling for day of the week and weather. To control for the potential confounding by weather, we included into the regression model average temperature for the same and previous day. Since risk may vary nonlinearly with temperature, we used a regression spline (with 3 degrees of freedom) both for the same and previous day. The effect of PM_2.5_ on hospitalization and mortality was modeled linearly.

Case-crossover analyses lend themselves to the analysis of effect modification. To determine whether the mortality risks associated with short-term PM_2.5_ among elderly is modified by a previous hospital admission of neurological disorders or diabetes, we included into the same regression model interaction terms between PM_2.5_ and the indicator variables indicating whether or not an individual had a primary or secondary hospitalization for diabetes, Parkinson’s disease, Alzheimer’s disease, and dementia.

To examine effect modification by sex, age (65–75, >75) or race (black, white, other races) we ran the conditional logistic regression stratified by each demographic characteristic. To compare the differences between the risk estimates in two categories of a potential effect modifier (e.g., the difference between males and females) we computed the 95 percent confidence interval as: Q^1-Q^2±1.96SE^12+SE^22 where and Q^1 and Q^2 are the estimates for the two categories (e.g., males and females), and SE1^ and SE2^ are their respective standard errors. We reject the null hypothesis that the group means are the same if the interval does not contain zero [52].

In a second stage of the analysis, the community specific results of the first stage analyses were combined using the multivariate meta-analysis technique of Berkey and coworkers [53]. To be conservative we report the results incorporating a random effect, whether or not there was a significant heterogeneity.

## Results

From the Medicare beneficiary denominator file we selected 6,982,678 deaths among all Medicare enrollees during the period 1999 to 2010 in 121 US communities. From the Medicare data we selected the subjects 65 and older who were hospitalized for the targeted medical conditions.

Table 1 shows the descriptive statistics for the counts of deaths, counts of previous cause-specific hospitalization among those who died; and the number of cause-specific hospital admissions summarized across the 121 communities. Among the subjects who died during the study period (approximately 7 million) in all the communities, 13.7%, 3.8%, and 3.3% of subjects had a previous hospitalization noting as a cause or a comorbid condition diabetes, Alzheimer’s disease, and dementia respectively. Parkinson's disease and multiple sclerosis represent a lower proportion of persons with this specific admission. The primary emergency cause-specific hospitalizations in the same table follow the same pattern, with the higher number of hospitalizations for diabetes, and Alzheimer’s disease, and very few primary admissions for multiple sclerosis.

**Table 1:** Counts of deaths among Medicare enrollees; counts of cause-specific admission prior to death; and counts of cause-specific primary emergency hospitalizations, summarized over the 121 cities

	**Minimum**	**1st Quartile**	**Mean**	**3rd Quartile**	**Maximum**	**Total**	**%**
**Deaths**	1,710	13,342	57,708	61,409	444,791	6,982,678	
**Previous cause-specific hospitalization among deaths:**							
Dementia	22	323	1,910	1,828	26,087	231,091	3.3
Alzheimer's disease	32	503	2,192	2,510	16,908	265,219	3.8
Parkinson’s disease	11	175	814	872	7,592	98,470	1.4
Multiple sclerosis	1	14	61	61	671	7,337	0.1
Diabetes	184	1,822	7,897	9,442	71,186	955,547	13.7
**Cause-specific hospitalizations:**							
Dementia	6	107	736	709	8,944	89,082	
Alzheimer's disease	10	252	1,208	1,530	9,846	146,172	
Parkinson’s disease	4	63	335	342	4,430	40,496	
Multiple sclerosis	0	7	38	38	563	4,634	
Diabetes	90	834	3,658	4,079	53,485	442,622	

**Figure 1:**
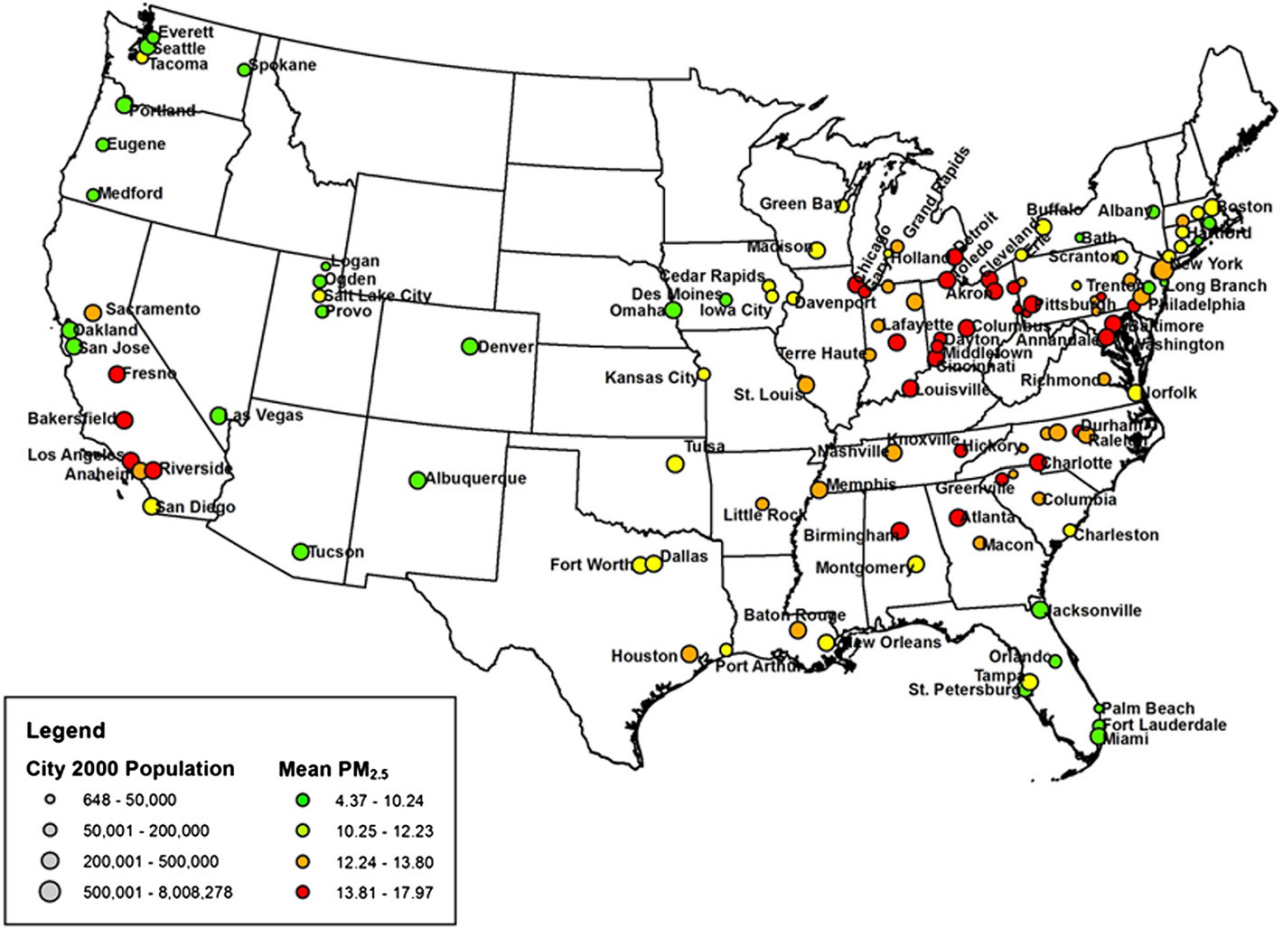
**Map of the US with the location of the 121 communities; the symbol size represent the population in each community, while the color represent the average PM**_**2.5 **_**in the community.**

Figure 1 shows the location of the 121 US communities included in the study; the symbol size represent the population in each community, while the color represent the average PM_2.5_ concentrations during the entire study period. High levels of PM_2.5_ (red) are in California and in the industrial Midwest.

Additional file 1: Table S1 presents for each of the 121 communities the daily mean concentration levels for PM_2.5_, and of temperature; the total number of deaths and total number of previous admissions before death for dementia, Alzheimer’s disease, Parkinson’s disease, multiple sclerosis and diabetes. Additional file 1: Table S2 shows the counts of primary emergency cause-specific hospitalizations. Due to the small number of primary admission for multiple sclerosis we excluded these from the hospitalization analysis.

Table 2 presents the results of the analyses for 1) all-cause mortality and 2) primary cause-specific hospital admissions. We found a 0.64% increase (95% CI: 0.42, 0.85) in all-cause mortality for each 10 μg/m^3^ increase in the two days average of PM_2.5_ among all Medicare participants. We also found 0.60% increase (95% CI: 0.36, 0.84) in all-cause mortality among persons who had never been hospitalized for the specified conditions during the study period. We also found a significant, and larger, association between short-term exposure to PM_2.5_ and all-cause mortality in subjects with previous hospital admissions for diabetes (0.76%; 0.39, 1.12), Parkinson’s disease (1.15%; 0.09, 2.23), dementia (0.94%; 0.01, 1.89), Alzheimer’s disease (1.04%; 0.36, 1.72). However these differences were not statistically significant.

**Table 2:** **Percent increase for 10 μg/m**^**3 **^**increase in the two days average PM**_**2.5**_**: Combined across the 121 communities**

		**%**	**95% CI**
**1) Mortality**			
All deaths	0.64	0.42	0.85
Deaths without medical conditions	0.60	0.36	0.84
**Mortality by previous cause of hospitalization**			
Alzheimer's disease	1.04	0.36	1.72
Dementia	0.94	0.01	1.89
Parkinson's disease	1.15	0.09	2.23
Multiple Sclerosis	4.01	-0.03	8.21
Diabetes	0.76	0.39	1.12
**2) Cause specific hospitalizations**			
Alzheimer's disease	0.20	-1.26	1.69
Dementia	0.92	-0.44	2.30
Parkinson's disease	3.23	1.08	5.43
Diabetes	1.14	0.56	1.73

1) Total Mortality in all Medicare Deaths and in subjects with previous cause-specific hospital admissions.

2) Cause-specific Primary Emergency Hospitalizations.

**Figure 2:**
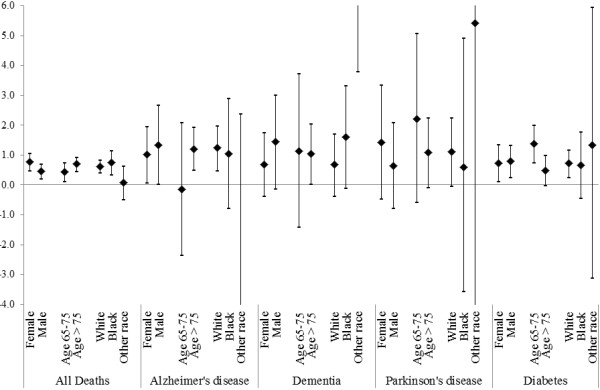
**Percent increase in total mortality for 10 μg/m**^**3 **^**increase in the two days average PM**_**2.5 **_**for all Medicare deaths and separately by specific previous cause of admission and by demographics characteristics.** The results are combined across the 121 communities.

**Figure 3:**
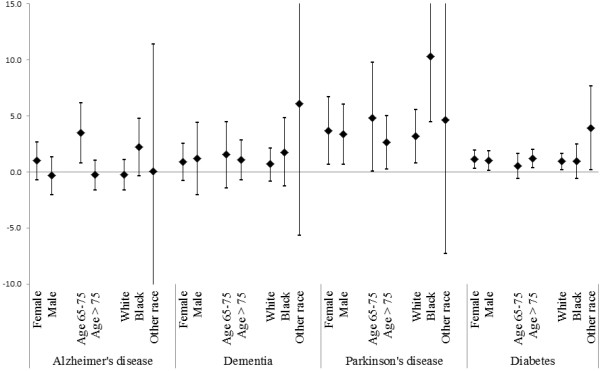
**Percent increase in cause specific primary emergency hospital admissions for 10 μg/m**^**3 **^**increase in the two days average PM**_**2.5 **_**separately by specific cause of admission and by demographics characteristics.** The results are combined across the 121 communities.

Table 2 summarizes also the results for cause-specific hospital admissions. For each 10 μg/m^3^ increase in the two days average of PM_2.5_ we found statistically significant increases in hospital admissions for Parkinson’s disease (3.23%; 95% CI: 1.08, 5.43), and diabetes (1.14%; 95% CI: 0.56, 1.73). The results for the other admissions causes are positive but non-significant due to the small number of cases in some cities, hence large confidence intervals in the meta-analysis.When the analysis was stratified by sex, race and age group we did not find significant evidence of effect modification by any of the demographic characteristics in the mortality analysis (Figure 2). In the hospital admissions analysis (Figure 3) instead we found that age was a significant modifier (P-value = 0.009) in Alzheimer’s disease with a 3.48% (95% CI: 0.83, 6.19) increase in subjects with an age between 65 and 75 years compared to a -0.27% (95% CI: -1.59, 1.06) increase in hospitalizations in subjects with over 75 years of age. The results by race are very unstable due to the distribution by race and cause of admissions.

**Figure 4:**
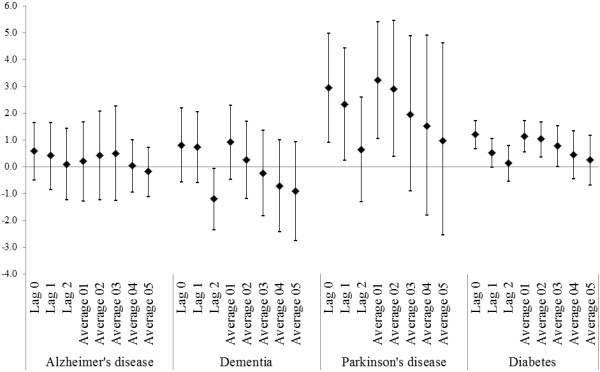
**Percent increase in cause specific primary emergency hospital admissions for 10 μg/m**^**3 **^**increase in PM**_**2.5 **_**for lags 0, 1, 2 and moving average from 2 days (01) up to 6 days (05).** The results are combined across the 121 communities.

Because of our novel association between PM_2.5_ and admissions for neurological disorders, we also examined the effect of up to 3 previous days (lags 0, 1, 2) and the cumulative effect expressed by the moving average of PM_2.5_ up to 6 previous days (moving average from 2 days (01) up to 6 days (05)) (Figure 4). We found that the effects are immediate at lag 0 and persist for 2 or 3 days averages of PM_2.5_.

## Discussion

Most early attention to the association of particles with hospitalization and mortality has focused on heart and lung disease; lately interest has shifted to cerebrovascular disease, stroke, and cognitive impairment. In this multi-city study we found that short term exposure to fine particles increase the risk of all-cause mortality and of hospitalizations for diabetes and neurological disorders.

This is the first study to examine susceptible population defined as those elderly with a hospitalization for dementia, Alzheimer’s disease, Parkinson’s disease, and multiple sclerosis.

We found significant effects of PM_2.5_ on hospitalization for Parkinson’s disease and diabetes; and in subjects aged 65–75 with Alzheimer’s disease. We also found that short term exposure to fine particles increased mortality risk in subjects with previous admissions for diabetes, Parkinson’s disease, dementia, and Alzheimer’s disease. While those risks were not significantly higher than the mortality risk associated with short-term effect of PM_2.5_ among then Medicare enrollees that were never hospitalized for any of these diseases, there was a pattern of higher estimates. The lack of significant effect modification of the mortality risk by pre-existing neurological disease could be due to limitation in the data used. More specifically, Medicare data only report hospitalization by cause and therefore we are not capturing enrollees with dementia or neurological disorder who are not hospitalized.

While this study focus on the mortality and hospitalizations of adults over the age of 65 with a specific admission for neurological disorder, previous studies have reported direct effects of particles on neurological outcomes including cognitive function. This suggests common pathways, which may explain the associations we saw.

In Germany elderly women were tested for mild cognitive impairment and the authors found that chronic exposure to traffic-related PM_2.5_ may be involved in the pathogenesis of Alzheimer’s disease [54]. Power and co-authors found that exposure to black carbon, a marker of traffic-related particles, was associated with worse cognitive function in older men [38]. In the Chinese Longitudinal Healthy Longevity Survey, the authors found that air pollution, measured by an index of particulate and gas concentrations, increased the odds of disability in activities of daily living, cognitive impairment, and health deficits [55]. Finally in a study of older women higher levels of long term exposure to PM_2.5_ and coarse particles were associated with significantly faster cognitive decline [56].

Air pollution can produce its adverse effects in the central nervous system (CNS), through a variety of cellular and molecular mechanisms [57]. Some hypotheses have been suggested to explain these observed associations. One hypothesis is that the presence of particles in the brain due to the migration of particles from the nose to the brain, can causes neurological disorders. An alternative hypothesis is that ambient particle inhalation has a primary effect on the vasculature in the central nervous system (CNS), resulting in subsequent impairment. Particles have been shown to translocate from the nose up the olfactory nerve into the brain, including the striatum frontal cortex, and cerebellum [58,59]. This in turn is associated with increased brain inflammatory responses [35,60] and changes in neurotransmitter levels [61]. In humans, diesel exhaust exposure has been shown to alter EEG responses, with patterns indicative of cortical stress [62]. In polluted areas of Mexico City, dogs had more prefrontal lesions, neuro-inflammation, gliosis, and particle deposition. In these polluted areas, brain MRI’s of children had more prefrontal lesions; autopsy studies of accident victims showed upregulation of cyclooxygenase-2 and CD14 [63-65].

Wellenius and co-authors [33] evaluated the association between PM_2.5_ and cerebrovascular hemodynamics in a cohort of older adults. The authors found that exposure to PM_2.5_ was associated with higher resting cerebrovascular resistance and lower cerebral blood flow velocity, suggesting that alterations in cerebrovascular hemodynamics may underlie the increased risk of particle-related acute cerebrovascular events.

Another component of vascular function is arterial stiffness; stiffening of the arteries is a process associated with aging and cardiovascular risk factors. In a recent paper Metha and co-authors [34], in a cohort of elderly men in the Boston area, found that short-term changes in air pollution were associated with augmentation index and augmentation pressure, correlates of arterial stiffness, supporting the hypothesis that exposure to air pollution may affect vascular function.

Therefore emerging evidence also suggests that possible biological mechanisms through which pollution can affect neurological disease are air pollution-induced neuro inflammation, oxidative stress, cerebrovascular dysfunction, and systemic inflammation. A better understanding of the mechanisms will enable the development of new strategies to protect individuals at risk and to reduce detrimental effects of air pollution on the nervous system and mental health [32,57].

We also found a significant PM_2.5_ effect in subjects with primary hospitalization for diabetes and in subjects with a previous admission for diabetes, which is higher (although not statistically significantly higher) than the mortality risk in subjects without any of the specified pre-existing conditions. Previous epidemiological studies suggested that people with a hospitalization for diabetes are vulnerable to cardiovascular health effects associated with exposure to particle air pollution. In a 2002 study of 4 US communities we found that diabetics have double the risk of a PM_10_-associated cardiovascular admission compared with non-diabetics [40]. Similarly, we estimated a 2.0-fold higher mortality risk associated with PM_10_ exposure for diabetics than for controls in a 2004 case-crossover study [41]. Likewise, PM_10_ effects on mortality were stronger in diabetics than in non-diabetics in 9 Italian communities [42]. O’Neill and co-authors in two studies examined Boston residents with type 2 diabetes. In one of the study [43] the authors found a positive association between air pollution and inflammatory markers as ICAM-1, VCAM-1 and vWF, suggesting that inflammatory mechanisms may explain the increased risk of air pollution-associated cardiovascular events among persons with diabetes. In the other study the authors found associations between air pollution and endothelium-dependent and -independent vascular reactivity measures [20]. Our results are not directly comparable to these previously published, but we also found increased mortality and hospitalizations risks in diabetics.

Similarly, studies have shown that air pollution is capable of causing these same physiological responses [15,37]. Some studies have suggested that PM may be associated with reduced heart rate variability [66-70], and increased C-reactive protein [24,25]. PM_2.5_ and PM_2.5_ components have also been shown to increase oxidative stress, including in the heart [24,26-28], to decrease plaque stability [29,30], and to increase atherosclerosis [31]. Therefore it is plausible for air pollution to be a factor in developing diabetes [71].

Among the limitations of the study, we measure “susceptibility” by a previous hospitalization as one of the diagnosis in a list of diseases. However, this clearly results in misclassification of the effect modifier, as people in our study population may have diabetes or dementia without a previous admission, or without it being noted in that admission. We also do not include Medicare participants in managed care because their care is paid for by capitation, and hence no specific events are reported to Medicare. This may limit generalization of these results. Another limitation though is due to Medicare reporting only the date of death and not the cause of death.

## Conclusions

In this multi-city study we found that short-term exposure to fine particles increased the risk of hospitalizations for Parkinson’s disease and diabetes and of all-cause mortality in Medicare enrollees. The associations persist in the mortality risk of subpopulations of Medicare patients with neurological disease and diabetes hospitalizations prior to death, even though these are not significantly different than the mortality risk among then Medicare enrollees that were never hospitalized for any of these diseases.

The same biological responses thought to effect cardiovascular disease through air pollution-mediated systemic oxidative stress, inflammation, and cerebrovascular dysfunction could also be relevant for diabetes and neurodegenerative diseases. Therefore these results give additional insight into the mechanisms by which particles may affect the brain. More studies are needed to understand the association between exposure and the risk of neurodegenerative diseases development, in order to develop preventative strategies, and improve air quality to reduce the burden of neurological morbidity and mortality.

## Abbreviations

PM_2.5_: Fine particulate air matter with aerodynamic diameter less than 2.5 microns; NO_2_: Nitrogen dioxide; MI: Myocardial Infarction; CI: Confidence Interval; MEDPAR: Medicare Provider Analysis and Review; ICD-9: International Classification of Diseases, Ninth Revision.; AD: Alzheimer’s disease; MS: Multiple sclerosis.

## Competing interests

The authors declare that they have no competing interests.

## Authors’ contributions

AZ: designed the study and conducted the analysis, interpreted the data, drafted the paper, and approved the final version. FD: obtained the data, interpreted the data, revised the paper critically for important intellectual content, and approved the final version. YW: created the dataset, conducted quality assurance and control, revised the paper, and approved the final version. JS: designed the study, interpreted the data, revised the paper, and approved the final version.

## Supplementary Material

Additional file 1: Table S1 and Table S2 A National analysis of the short term effect of PM_2.5_ on hospitalizations and mortality in subjects with diabetes and neurological disorders. Click here for file
