# Supplementary material for: A national case-crossover analysis of the short-term effect of PM2.5 on hospitalizations and mortality in subjects with diabetes and neurological disorders
Source: Environ Health. 2014 May 22;13:38. doi: 10.1186/1476-069X-13-38 (PMC4064518; doi:10.1186/1476-069X-13-38)
Supplement: Additional file 1: Table S1 and Table S2 — A National analysis of the short term effect of PM2.5 on hospitalizations and mortality in subjects with diabetes and neurological disorders. [file 1476-069X-13-38-S1.docx]

**Additional file 1**

**A National analysis of the short term effect of PM_2.5_ on hospitalizations and mortality in subjects with diabetes and neurological disorders**

| **Supplemental Table 1: Community specific average PM2.5 and temperature; and total number of deaths** | | | | | | | | | |
| --- | --- | --- | --- | --- | --- | --- | --- | --- | --- |
| **and previous cause specific admissions** | | |  |  |  |  |  |  |  |
| STATE | CITY | PM25 | Temperature |  | Previous admissions among Medicare deaths | | | | |
|  |  | mg/m3 | F | Total | Dementia | Alzheimer's | Parkinson's | Multiple | Diabetes |
|  |  | Mean | Mean | deaths |  | disease | disease | sclerosis |  |
| Alabama | Birmingham | 15.1 | 63.4 | 79631 | 3202 | 3949 | 1167 | 58 | 11090 |
| Alabama | Montgomery | 11.9 | 65.6 | 5399 | 196 | 252 | 60 | 1 | 836 |
| Arizona | Tucson | 6.1 | 70.6 | 27784 | 480 | 888 | 304 | 16 | 2225 |
| Arkansas | Little Rock | 13.2 | 62.7 | 23848 | 1397 | 1284 | 352 | 26 | 3800 |
| California | Oakland | 8.8 | 56.4 | 45439 | 900 | 1086 | 404 | 28 | 3955 |
| California | Fresno | 18.0 | 64.4 | 46894 | 914 | 1946 | 619 | 31 | 6604 |
| California | Bakersfield | 16.4 | 65.5 | 40351 | 750 | 1336 | 446 | 24 | 4511 |
| California | Los Angeles | 16.0 | 62.2 | 444791 | 13408 | 11777 | 5323 | 319 | 52354 |
| California | Anaheim | 13.8 | 64.1 | 139814 | 3976 | 3811 | 1519 | 111 | 12577 |
| California | Riverside | 15.4 | 64.0 | 190665 | 3134 | 4274 | 1447 | 111 | 16081 |
| California | Sacramento | 12.6 | 60.1 | 58195 | 1306 | 1246 | 565 | 32 | 4743 |
| California | San Diego | 11.8 | 62.2 | 174016 | 3155 | 4427 | 1517 | 147 | 13863 |
| California | San Jose | 9.7 | 63.4 | 26803 | 445 | 650 | 263 | 16 | 2488 |
| Colorado | Denver | 9.7 | 50.8 | 76351 | 1009 | 1637 | 611 | 72 | 5092 |
| Connecticut | Stamford | 10.3 | 52.1 | 20265 | 729 | 928 | 242 | 28 | 2813 |
| Connecticut | Hartford | 10.6 | 52.2 | 73895 | 2515 | 2911 | 1005 | 88 | 10944 |
| Connecticut | New Haven | 12.2 | 52.3 | 64881 | 2092 | 2616 | 1112 | 84 | 10034 |
| Connecticut | New London | 9.1 | 51.5 | 6853 | 128 | 210 | 58 | 9 | 1206 |
| Delaware | Wilmington | 13.9 | 55.2 | 34876 | 1135 | 1500 | 487 | 43 | 5731 |
| District Of Columbia | Washington | 13.8 | 58.2 | 47427 | 1828 | 2099 | 587 | 50 | 8492 |
| Florida | Fort Lauderdale | 7.9 | 76.5 | 135903 | 2393 | 4350 | 1953 | 117 | 14074 |
| Florida | Jacksonville | 9.8 | 70.1 | 48367 | 1358 | 2330 | 654 | 43 | 8059 |
| Florida | Tampa | 10.3 | 72.7 | 61387 | 1873 | 2276 | 736 | 47 | 7848 |
| Florida | Miami | 9.4 | 76.4 | 53070 | 1635 | 2289 | 970 | 31 | 7579 |
| Florida | Orlando | 9.3 | 72.1 | 74768 | 1868 | 3469 | 1046 | 58 | 11795 |
| Florida | Palm Beach | 7.4 | 75.2 | 126264 | 3182 | 4130 | 2087 | 129 | 14545 |
| Florida | St. Petersburg | 9.6 | 73.7 | 110157 | 2941 | 3727 | 1287 | 98 | 11945 |
| Georgia | Macon | 12.8 | 63.9 | 4551 | 139 | 205 | 49 | 4 | 803 |
| Georgia | Atlanta | 15.8 | 61.8 | 130351 | 3540 | 7551 | 1863 | 138 | 18914 |
| Hawaii | Honolulu | 4.4 | 77.2 | 56699 | 1643 | 1224 | 554 | 14 | 6042 |
| Illinois | Chicago | 14.1 | 52.3 | 401172 | 15085 | 16908 | 7592 | 671 | 71186 |
| Indiana | Fort Wayne | 13.2 | 50.9 | 7670 | 154 | 280 | 78 | 10 | 963 |
| Indiana | Gary | 15.7 | 53.9 | 13452 | 698 | 611 | 276 | 15 | 2734 |
| Indiana | Indianapolis | 15.3 | 53.8 | 60364 | 3063 | 2972 | 796 | 65 | 10073 |
| Indiana | South Bend | 12.8 | 50.1 | 7164 | 217 | 281 | 73 | 10 | 1062 |
| Indiana | Lafayette | 12.5 | 52.5 | 3015 | 64 | 162 | 27 | 6 | 497 |
| Indiana | Terre Haute | 12.9 | 53.1 | 3096 | 57 | 187 | 44 | 1 | 622 |
| Iowa | Iowa city | 11.2 | 49.5 | 1710 | 40 | 32 | 21 | 2 | 184 |
| Iowa | Cedar Rapids | 10.8 | 48.5 | 13342 | 276 | 496 | 175 | 22 | 1961 |
| Iowa | Des Moines | 10.2 | 51.2 | 23940 | 382 | 984 | 385 | 35 | 3701 |
| Illinois | Davenport | 12.1 | 49.9 | 23143 | 415 | 871 | 440 | 33 | 3775 |
| Kansas | Kansas | 11.5 | 56.8 | 104286 | 3186 | 5085 | 1688 | 124 | 13946 |
| Kentucky | Louisville | 14.8 | 58.4 | 59789 | 1807 | 2833 | 1178 | 61 | 10041 |
| Louisiana | Baton Rouge | 12.6 | 67.9 | 27520 | 565 | 1601 | 418 | 11 | 4326 |
| Louisiana | New Orleans | 11.6 | 71.4 | 61409 | 1796 | 2510 | 732 | 34 | 8302 |
| Maryland | Marlboro | 11.0 | 56.3 | 8930 | 267 | 315 | 97 | 4 | 1822 |
| Maryland | Baltimore | 14.1 | 58.3 | 109533 | 3401 | 3795 | 1439 | 136 | 18045 |
| Massachusetts | Springfield | 12.3 | 50.7 | 17218 | 452 | 534 | 259 | 17 | 2304 |
| Massachusetts | Boston | 11.0 | 51.6 | 182121 | 8926 | 6962 | 2438 | 278 | 23666 |
| Massachusetts | Worcester | 11.3 | 47.8 | 20667 | 954 | 529 | 271 | 14 | 2048 |
| Michigan | Holland | 12.1 | 49.2 | 3458 | 127 | 138 | 71 | 4 | 546 |
| Michigan | Grand Rapids | 13.6 | 48.8 | 21841 | 902 | 788 | 363 | 41 | 3156 |
| Michigan | Detroit | 14.1 | 51.2 | 303502 | 15589 | 13917 | 5179 | 511 | 55700 |
| Missouri | St. Louis | 13.8 | 56.3 | 130262 | 5272 | 5731 | 2066 | 141 | 17852 |
| Nebraska | Omaha | 10.0 | 51.9 | 28658 | 863 | 1106 | 392 | 46 | 3932 |
| Nevada | Las Vegas | 7.5 | 69.9 | 59585 | 911 | 1414 | 616 | 49 | 5665 |
| New Jersey | Jersey city | 11.4 | 54.4 | 10706 | 538 | 587 | 149 | 10 | 2380 |
| New Jersey | Trenton | 9.4 | 54.9 | 9376 | 198 | 607 | 128 | 16 | 1790 |
| New Jersey | Long Branch | 8.8 | 53.9 | 22146 | 419 | 1303 | 218 | 32 | 3931 |
| New Jersey | Elizabeth | 13.3 | 55.4 | 36484 | 1611 | 2305 | 687 | 42 | 6676 |
| New Mexico | Albuquerque | 6.5 | 58.0 | 36779 | 433 | 567 | 302 | 34 | 2388 |
| New York | Albany | 8.7 | 49.2 | 7680 | 322 | 311 | 76 | 3 | 941 |
| New York | Buffalo | 10.3 | 48.3 | 28565 | 540 | 464 | 194 | 24 | 2467 |
| New York | New York | 13.4 | 51.3 | 431647 | 26087 | 14443 | 7316 | 405 | 67946 |
| New York | Bath | 8.7 | 48.8 | 8353 | 270 | 376 | 130 | 13 | 1565 |
| North Carolina | Hickory | 12.6 | 58.8 | 4237 | 109 | 160 | 57 | 4 | 630 |
| North Carolina | Durham | 14.3 | 60.0 | 9478 | 609 | 392 | 137 | 10 | 1569 |
| North Carolina | Winston-Salem | 13.6 | 59.5 | 25396 | 758 | 1103 | 293 | 25 | 3281 |
| North Carolina | Greensboro | 13.2 | 59.3 | 30333 | 807 | 1293 | 374 | 34 | 4043 |
| North Carolina | Charlotte | 14.0 | 60.7 | 38487 | 1339 | 2097 | 544 | 40 | 6139 |
| North Carolina | Raleigh | 13.2 | 60.5 | 32110 | 1405 | 1604 | 459 | 28 | 5001 |
| Ohio | Middletown | 16.5 | 53.1 | 9683 | 248 | 424 | 177 | 5 | 1568 |
| Ohio | Youngstown | 15.4 | 49.7 | 25554 | 770 | 1442 | 459 | 47 | 4584 |
| Ohio | Cleveland | 15.5 | 52.5 | 89451 | 3745 | 3720 | 1607 | 156 | 13847 |
| Ohio | Columbus | 16.2 | 53.5 | 34637 | 823 | 1441 | 594 | 48 | 5318 |
| Ohio | Cincinnati | 16.1 | 54.7 | 71060 | 1808 | 2359 | 930 | 73 | 9535 |
| Ohio | Steubenville | 17.1 | 52.0 | 6965 | 314 | 488 | 171 | 5 | 1443 |
| Ohio | Toledo | 14.9 | 50.8 | 16395 | 465 | 686 | 269 | 18 | 2548 |
| Ohio | Dayton | 16.3 | 53.2 | 19679 | 1023 | 738 | 368 | 14 | 2920 |
| Ohio | Akron | 16.0 | 50.6 | 24201 | 1068 | 1091 | 387 | 27 | 3605 |
| Oklahoma | Tulsa | 11.4 | 61.6 | 22917 | 653 | 913 | 259 | 21 | 2901 |
| Oregon | Medford | 10.0 | 54.6 | 5862 | 236 | 170 | 96 | 5 | 612 |
| Oregon | Eugene | 8.8 | 52.2 | 26706 | 685 | 670 | 240 | 29 | 2430 |
| Oregon | Portland | 9.0 | 53.5 | 32704 | 803 | 639 | 280 | 33 | 1990 |
| Pennsylvania | Gettysburg | 12.6 | 53.0 | 7449 | 165 | 297 | 91 | 8 | 1277 |
| Pennsylvania | Pittsburgh | 15.0 | 52.1 | 135684 | 3721 | 4443 | 2193 | 137 | 15770 |
| Pennsylvania | State College | 11.8 | 50.5 | 7171 | 170 | 199 | 111 | 8 | 1080 |
| Pennsylvania | Carlisle | 13.7 | 54.1 | 18849 | 363 | 912 | 359 | 22 | 3001 |
| Pennsylvania | Harrisburg | 14.4 | 54.3 | 21661 | 391 | 1067 | 385 | 31 | 3576 |
| Pennsylvania | Erie | 12.0 | 50.3 | 20743 | 322 | 503 | 156 | 26 | 2503 |
| Pennsylvania | Scranton | 11.4 | 50.1 | 65063 | 1932 | 2936 | 1386 | 64 | 11260 |
| Pennsylvania | Allentown | 13.2 | 52.3 | 54645 | 1293 | 2264 | 872 | 63 | 9110 |
| Pennsylvania | Mercer | 12.8 | 49.3 | 10820 | 214 | 537 | 116 | 9 | 1651 |
| New Jersey | Philadelphia | 13.3 | 56.4 | 385916 | 13730 | 15346 | 5616 | 407 | 50650 |
| Pennsylvania | Washington | 13.9 | 50.9 | 23250 | 681 | 820 | 487 | 22 | 3156 |
| Rhode Island | Providence | 10.2 | 52.1 | 69266 | 1780 | 2104 | 749 | 81 | 7574 |
| South Carolina | Charleston | 11.3 | 65.5 | 21638 | 672 | 1090 | 287 | 11 | 3701 |
| South Carolina | Greenville | 14.0 | 61.5 | 27191 | 739 | 1597 | 341 | 16 | 4234 |
| South Carolina | Columbia | 13.0 | 64.2 | 26414 | 687 | 1222 | 320 | 23 | 4298 |
| South Carolina | Spartanburg | 13.4 | 61.0 | 21058 | 1069 | 1321 | 385 | 19 | 3414 |
| Tennessee | Nashville | 12.8 | 60.2 | 33386 | 1619 | 1486 | 454 | 24 | 4607 |
| Tennessee | Knoxville | 14.2 | 59.3 | 34220 | 1449 | 1744 | 468 | 27 | 5449 |
| Tennessee | Memphis | 12.3 | 63.4 | 47959 | 1895 | 2937 | 717 | 37 | 9442 |
| Texas | Dallas | 11.8 | 67.4 | 107842 | 3403 | 5343 | 1626 | 105 | 16250 |
| Texas | Houston | 12.6 | 70.1 | 142137 | 4428 | 6534 | 2164 | 137 | 22851 |
| Texas | Port Arthur | 11.1 | 68.7 | 9209 | 220 | 621 | 145 | 6 | 1815 |
| Texas | Ft Worth | 11.9 | 66.1 | 51588 | 1535 | 2319 | 867 | 39 | 6787 |
| Utah | Logan | 9.7 | 46.1 | 2867 | 100 | 60 | 25 | 6 | 284 |
| Utah | Salt Lake | 11.0 | 53.3 | 42693 | 872 | 1093 | 486 | 59 | 4455 |
| Utah | Provo | 9.4 | 50.9 | 14342 | 237 | 393 | 193 | 17 | 1714 |
| Utah | Ogden | 9.3 | 50.6 | 2976 | 22 | 44 | 11 | 5 | 283 |
| Virginia | Annandale | 12.9 | 57.9 | 38357 | 938 | 1412 | 597 | 41 | 3731 |
| Virginia | Richmond | 12.7 | 59.1 | 50790 | 1203 | 2263 | 746 | 43 | 7856 |
| Virginia | Norfolk | 11.8 | 60.7 | 80895 | 3771 | 3698 | 959 | 95 | 14544 |
| Washington | Vancouver | 8.0 | 53.7 | 7668 | 121 | 182 | 75 | 11 | 716 |
| Washington | Seattle | 9.1 | 51.8 | 103329 | 3217 | 3101 | 1217 | 159 | 9892 |
| Washington | Tacoma | 11.4 | 51.2 | 14790 | 605 | 494 | 206 | 18 | 1710 |
| Washington | Everett | 7.8 | 50.1 | 12252 | 183 | 283 | 82 | 12 | 1110 |
| Washington | Spokane | 8.9 | 48.8 | 11076 | 323 | 325 | 161 | 18 | 1180 |
| Wisconsin | Green Bay | 11.3 | 45.8 | 4954 | 68 | 157 | 50 | 3 | 570 |
| Wisconsin | Madison | 11.8 | 47.3 | 8397 | 127 | 259 | 65 | 13 | 846 |

| **Supplemental Table 2: Community specific cause-specific primary emergency hospitalizations** | | | | | | |
| --- | --- | --- | --- | --- | --- | --- |
|  |  |  |  |  |  |  |
| STATE | CITY | Primary Emergency Hospital Admissions | | | | |
|  |  | Dementia | Alzheimer's | Parkinson's | Multiple | Diabetes |
|  |  |  | disease | disease | sclerosis |  |
| Alabama | Birmingham | 2350 | 3866 | 446 | 58 | 4172 |
| Alabama | Montgomery | 60 | 311 | 20 | 1 | 333 |
| Arizona | Tucson | 213 | 613 | 157 | 16 | 1205 |
| Arkansas | Little Rock | 1360 | 2229 | 111 | 13 | 1847 |
| California | Oakland | 140 | 504 | 136 | 25 | 1726 |
| California | Fresno | 142 | 321 | 123 | 8 | 2962 |
| California | Bakersfield | 105 | 237 | 91 | 28 | 2189 |
| California | Los Angeles | 114 | 305 | 136 | 15 | 1693 |
| California | Anaheim | 709 | 2219 | 571 | 35 | 6132 |
| California | Riverside | 501 | 1237 | 472 | 41 | 8301 |
| California | Sacramento | 192 | 330 | 146 | 9 | 1893 |
| California | San Diego | 684 | 1606 | 585 | 59 | 6508 |
| California | San Jose | 54 | 162 | 63 | 13 | 1022 |
| Colorado | Denver | 422 | 1016 | 296 | 32 | 2580 |
| Connecticut | Stamford | 196 | 493 | 117 | 38 | 1143 |
| Connecticut | Hartford | 1184 | 1772 | 266 | 37 | 4445 |
| Connecticut | New Haven | 872 | 1857 | 342 | 51 | 4707 |
| Connecticut | New London | 65 | 189 | 35 | 7 | 389 |
| Delaware | Wilmington | 299 | 634 | 210 | 45 | 2591 |
| District Of Columbia | Washington | 546 | 985 | 281 | 50 | 5762 |
| Florida | Fort Lauderdale | 579 | 1284 | 649 | 65 | 6693 |
| Florida | Jacksonville | 393 | 999 | 283 | 34 | 4293 |
| Florida | Tampa | 491 | 1148 | 309 | 25 | 3417 |
| Florida | Miami | 249 | 472 | 277 | 38 | 4379 |
| Florida | Orlando | 526 | 1530 | 545 | 44 | 5896 |
| Florida | Palm Beach | 553 | 1105 | 672 | 99 | 5395 |
| Florida | St. Petersburg | 1035 | 2164 | 581 | 43 | 4994 |
| Georgia | Macon | 28 | 48 | 17 | 3 | 333 |
| Georgia | Atlanta | 1843 | 3859 | 707 | 78 | 8285 |
| Hawaii | Honolulu | 375 | 373 | 169 | 6 | 2618 |
| Illinois | Chicago | 5584 | 9846 | 4430 | 563 | 40915 |
| Indiana | Fort Wayne | 173 | 220 | 20 | 8 | 316 |
| Indiana | Gary | 268 | 390 | 141 | 20 | 1863 |
| Indiana | Indianapolis | 1345 | 3421 | 475 | 68 | 4576 |
| Indiana | South Bend | 43 | 97 | 27 | 7 | 304 |
| Indiana | Lafayette | 21 | 210 | 12 | 1 | 170 |
| Indiana | Terre Haute | 31 | 97 | 18 | 1 | 190 |
| Iowa | Iowa city | 17 | 31 | 23 | 1 | 90 |
| Iowa | Cedar Rapids | 126 | 458 | 51 | 3 | 641 |
| Iowa | Des Moines | 158 | 493 | 163 | 19 | 1304 |
| Illinois | Davenport | 151 | 450 | 203 | 13 | 1301 |
| Kansas | Kansas | 2495 | 3033 | 762 | 81 | 6525 |
| Kentucky | Louisville | 842 | 2813 | 407 | 21 | 3343 |
| Louisiana | Baton Rouge | 180 | 405 | 131 | 5 | 1741 |
| Louisiana | New Orleans | 722 | 1408 | 187 | 9 | 3647 |
| Maryland | Marlboro | 56 | 214 | 47 | 3 | 1073 |
| Maryland | Baltimore | 1254 | 2383 | 869 | 102 | 9910 |
| Massachusetts | Springfield | 476 | 392 | 99 | 12 | 1088 |
| Massachusetts | Boston | 5352 | 6296 | 1674 | 173 | 12158 |
| Massachusetts | Worcester | 823 | 286 | 131 | 3 | 844 |
| Michigan | Holland | 102 | 70 | 20 | 0 | 194 |
| Michigan | Grand Rapids | 511 | 252 | 96 | 11 | 990 |
| Michigan | Detroit | 4702 | 7510 | 2198 | 431 | 25899 |
| Missouri | St. Louis | 2271 | 5126 | 1038 | 102 | 8562 |
| Nebraska | Omaha | 351 | 581 | 199 | 17 | 1553 |
| Nevada | Las Vegas | 277 | 527 | 219 | 17 | 2514 |
| New Jersey | Jersey city | 223 | 484 | 85 | 7 | 1703 |
| New Jersey | Trenton | 54 | 247 | 61 | 15 | 890 |
| New Jersey | Long Branch | 72 | 589 | 121 | 22 | 1632 |
| New Jersey | Elizabeth | 296 | 977 | 229 | 19 | 3596 |
| New Mexico | Albuquerque | 221 | 317 | 103 | 6 | 1022 |
| New York | Albany | 23 | 134 | 34 | 3 | 387 |
| New York | Buffalo | 107 | 271 | 107 | 17 | 1012 |
| New York | New York | 8944 | 9743 | 3557 | 324 | 53485 |
| New York | Bath | 81 | 171 | 36 | 4 | 715 |
| North Carolina | Hickory | 42 | 104 | 16 | 0 | 181 |
| North Carolina | Durham | 230 | 167 | 44 | 8 | 885 |
| North Carolina | Winston-Salem | 171 | 447 | 124 | 20 | 1277 |
| North Carolina | Greensboro | 187 | 536 | 146 | 18 | 1672 |
| North Carolina | Charlotte | 367 | 1177 | 198 | 37 | 2772 |
| North Carolina | Raleigh | 319 | 601 | 217 | 51 | 2125 |
| Ohio | Middletown | 181 | 232 | 62 | 0 | 483 |
| Ohio | Youngstown | 439 | 894 | 183 | 11 | 1615 |
| Ohio | Cleveland | 3766 | 3322 | 808 | 90 | 6602 |
| Ohio | Columbus | 364 | 516 | 191 | 26 | 2113 |
| Ohio | Cincinnati | 1395 | 1625 | 408 | 30 | 3921 |
| Ohio | Steubenville | 62 | 218 | 76 | 12 | 637 |
| Ohio | Toledo | 267 | 414 | 138 | 29 | 1039 |
| Ohio | Dayton | 498 | 393 | 168 | 20 | 1301 |
| Ohio | Akron | 642 | 531 | 168 | 12 | 1432 |
| Oklahoma | Tulsa | 324 | 708 | 77 | 10 | 1108 |
| Oregon | Medford | 76 | 57 | 31 | 3 | 203 |
| Oregon | Eugene | 250 | 295 | 59 | 15 | 668 |
| Oregon | Portland | 303 | 428 | 103 | 11 | 744 |
| Pennsylvania | Gettysburg | 70 | 209 | 44 | 11 | 405 |
| Pennsylvania | Pittsburgh | 1935 | 2311 | 1138 | 80 | 6924 |
| Pennsylvania | State College | 40 | 160 | 47 | 5 | 295 |
| Pennsylvania | Carlisle | 119 | 414 | 182 | 17 | 834 |
| Pennsylvania | Harrisburg | 190 | 527 | 185 | 8 | 1269 |
| Pennsylvania | Erie | 129 | 350 | 93 | 29 | 981 |
| Pennsylvania | Scranton | 882 | 1773 | 698 | 40 | 4079 |
| Pennsylvania | Allentown | 467 | 1089 | 415 | 23 | 4196 |
| Pennsylvania | Mercer | 46 | 347 | 76 | 5 | 598 |
| New Jersey | Philadelphia | 5506 | 7759 | 2341 | 289 | 26185 |
| Pennsylvania | Washington | 408 | 385 | 172 | 12 | 1283 |
| Rhode Island | Providence | 887 | 1762 | 345 | 30 | 3359 |
| South Carolina | Charleston | 288 | 434 | 102 | 9 | 1948 |
| South Carolina | Greenville | 316 | 737 | 107 | 6 | 1450 |
| South Carolina | Columbia | 222 | 863 | 102 | 4 | 1837 |
| South Carolina | Spartanburg | 396 | 899 | 102 | 12 | 1212 |
| Tennessee | Nashville | 1316 | 1523 | 185 | 12 | 1937 |
| Tennessee | Knoxville | 1116 | 2547 | 231 | 24 | 1653 |
| Tennessee | Memphis | 751 | 2526 | 280 | 25 | 4429 |
| Texas | Dallas | 1500 | 2203 | 600 | 59 | 7751 |
| Texas | Houston | 1037 | 2119 | 753 | 84 | 11901 |
| Texas | Port Arthur | 54 | 377 | 36 | 1 | 1054 |
| Texas | Ft Worth | 485 | 586 | 269 | 20 | 2817 |
| Utah | Logan | 6 | 10 | 11 | 0 | 95 |
| Utah | Salt Lake | 236 | 460 | 188 | 22 | 1643 |
| Utah | Provo | 61 | 97 | 40 | 12 | 589 |
| Utah | Ogden | 15 | 43 | 4 | 0 | 93 |
| Virginia | Annandale | 219 | 612 | 217 | 41 | 1670 |
| Virginia | Richmond | 524 | 1621 | 354 | 57 | 3436 |
| Virginia | Norfolk | 2099 | 2097 | 446 | 69 | 6515 |
| Washington | Vancouver | 37 | 83 | 24 | 2 | 199 |
| Washington | Seattle | 2616 | 2014 | 472 | 69 | 3274 |
| Washington | Tacoma | 370 | 198 | 71 | 8 | 459 |
| Washington | Everett | 78 | 223 | 24 | 2 | 337 |
| Washington | Spokane | 97 | 176 | 55 | 7 | 436 |
| Wisconsin | Green Bay | 21 | 51 | 25 | 6 | 274 |
| Wisconsin | Madison | 28 | 112 | 59 | 7 | 371 |
